# Association of triglycerides/high-density lipoprotein ratio with cardiometabolic risk factors in children from mothers who had gestational diabetes

**DOI:** 10.1590/1984-0462/2025/43/2025131

**Published:** 2025-12-12

**Authors:** Laura Bellini Alves de Souza, Leonardo Duarte Sobreira Luna, Micaela Frasson Montero, Filipe Dias de Souza, Martha Camillo Jordão, Maria Carolina Oliveira Babate, Rosiane Mattar, Sergio Atala Dib, Patricia Medici Dualib, Bianca de Almeida-Pititto

**Affiliations:** a Universidade Federal de São Paulo, São Paulo, SP, Brasil.

**Keywords:** Gestational diabetes, Cholesterol, HDL, Triglycerides, Children, Adolescents, Cardiometabolic risk factors, Diabetes gestacional, HDL-colesterol, Triglicerídeos, Crianças, Adolescentes, Fatores de risco cardiometabólico

## Abstract

**Objective:**

The aim of the study was to evaluate the association between triglycerides/high-density lipoprotein-cholesterol (TG/HDL) ratio and cardiometabolic risk factors in children of mothers who had gestational diabetes (GDM).

**Methods:**

This retrospective cohort study involved 227 offspring aged 2–14 years, born to mothers with GDM. Data was collected during routine antenatal care, and the offspring were evaluated 2–14 years later. TG/HDL ratio was stratified into tertiles. Cardiometabolic risk factors were defined according to the World Health Organization (WHO) criteria for children: obesity, overweight, elevated waist circumference, elevated blood pressure and hypertension, dysglycemia, low HDL-cholesterol, and elevated TG. The association of TG/HDL ratio and cardiometabolic risk factors was evaluated through comparative and linear regression analysis.

**Results:**

There were higher mean levels of some markers of cardiometabolic risk factors according to TG/HDL ratio tertiles. We observed higher frequencies of elevated HbA1c from 2 to 4 years (0 vs. 3.7 vs. 12.9%, p-linear-by-linear <0.001); higher prevalence of overweight/obesity from 5 to 9 years (39.1 vs. 47.2 vs. 75.0%, p-linear-by-linear<0.001), and higher frequencies of elevated low density lipoprotein-cholesterol from 10 to 14 years (33.3 vs. 66.6 vs. 77.7%, p-linear-by-linear=0.044) according to TG/HDL ratio tertiles. TG/HDL ratio was associated with homeostasis model assessment-insulin resistance from 5 to 9 years (β0.11 [95% confidence interval (95%CI) 0.00–0.23]) and from 10 to 14 years (β0.10 [95%CI 0.02–0.18]), after adjustments for sex and body mass index.

**Conclusions:**

Our results show that higher levels of TG/HDL ratio are associated with worse cardiometabolic profile and insulin resistance in offspring from mothers who had gestational diabetes mellitus and can be a good marker to identify those at higher cardiometabolic risk.

## RESUMO

**Objetivo:**

Avaliar a associação entre a razão triglicerídeos/HDL-colesterol (TG/HDL) e fatores de risco cardiometabólicos em filhos de mães que tiveram diabetes *mellitus* gestacional (DMG).

**Métodos:**

Esta coorte retrospectiva envolveu 227 crianças com idades entre dois e 14 anos, filhos de mães que tiveram DMG. Os dados foram coletados durante o acompanhamento pré-natal de rotina e os filhos foram avaliados de dois a 14 anos depois. A razão TG/HDL foi estratificada em tercis: de dois a nove anos, 1,2 e 2,0; de dez a 14 anos, 1,4 e 3,0. Os fatores de risco cardiometabólicos foram definidos de acordo com os critérios da Organização Mundial da Saúde (OMS) e das faixas etárias: obesidade, sobrepeso; circunferência abdominal elevada; pressão arterial elevada e hipertensão; disglicemia; HDL-colesterol baixo e triglicerídeos elevados. A associação entre a razão TG/HDL e os fatores de risco cardiometabólicos foi avaliada por meio de análises comparativas e de regressão linear.

**Resultados:**

Foram observados níveis mais elevados de marcadores de fatores de risco cardiometabólicos de acordo com os tercis da razão TG/HDL. Observou-se maior frequência de HbA1c elevada entre dois e quatro anos (0*vs.*3,7*vs.*12,9%, *p* linear-by-linear<0,001); maior prevalência de sobrepeso/obesidade entre cinco e nove anos (39,1*vs*.47,2*vs*.75%, *p* linear-by-linear<0,001); e maiores frequências de LDL-colesterol elevado entre dez e 14 anos (33,3*vs*.66,6*vs*.77,7%, *p* linear-by-linear=0,044), de acordo com os tercis da razão TG/HDL. A razão TG/HDL foi associada ao HOMA-IR entre cinco e nove anos [β=0,11 (intervalo de confiança de 95% — IC95% 0,00–0,23)] e entre dez e 14 anos [β=0,10 (IC95% 0,02–0,18)], após ajustes para sexo e índice de massa corpórea (IMC).

**Conclusões:**

Nossos resultados mostram que níveis elevados da razão TG/HDL estão associados a piores perfis cardiometabólicos e à resistência à insulina em filhos de mães que tiveram DMG, podendo ser bons marcadores para identificar aqueles com maior risco cardiometabólico.

## INTRODUCTION

There is great concern regarding the rise in obesity and worsening cardiometabolic profiles in children in Brazil and worldwide. In 2019, the Cardiovascular Risk Study in Adolescents (ERICA) evaluated over 70,000 adolescents aged 12–17 years and found that 25.5% were overweight/obese, 9.6% had elevated blood pressure, 47.3% had low high-density lipoprotein (HDL)-cholesterol levels, and more than half considered themselves sedentary, factors reflecting morbidity prevalence and a worse cardiometabolic profile.^1^ This aligns with global data: according to World Health Organization (WHO), the prevalence of overweight and obesity in youth aged 5–19 years rose from 8% in 1990 to 20% in 2022.^2^

Efforts are underway to identify at-risk youth early to enable preventive interventions and improve long-term health. Understanding the risk factors for childhood obesity and related morbidities is key. One such factor is gestational diabetes mellitus (GDM), which increases the mother's risk of type 2 diabetes and cardiovascular disease, and the offspring's risk of obesity, diabetes, dyslipidemia, and hypertension.^3^ GDM's high prevalence, affecting 3–25% of pregnancies depending on ethnicity and diagnostic criteria, makes it a relevant concern.^4^

The triglycerides/HDL-cholesterol (TG/HDL) ratio has recently emerged as a good marker for cardiometabolic risk and insulin resistance.^5-7^ It is low-cost, accurate, and routinely measured.^5^ Among children and adolescents, it has been used to assess insulin resistance in obese^5^ and overweight youth^6^ and as a potential marker for metabolic syndrome.^7^ However, these studies rarely include children under 10 or those born to mothers with GDM.

Therefore, it is important to evaluate the TG/HDL ratio in children of mothers with GDM, including those who are not obese, starting from age 2, to identify early signs of adverse cardiometabolic profiles. This study aims to examine the association of TG/HDL ratio with cardiometabolic risk factors in children aged 2–14 years born to mothers with GDM.

## METHOD

The study is a cross-sectional analysis of the children of mothers who had GDM, which is part of a larger retrospective cohort study previously approved by the Ethics and Research Committee of Universidade Federal de São Paulo (CAAE: 35470320.1.0000.5505). The current study represents a database analysis of this larger study, using the data from the current in-person collection of the children of mothers who had GDM.

Maternal data related to the gestational period were collected throughout the follow-up of these women at the Diabetes and Pregnancy outpatient clinic at Universidade Federal de São Paulo (UNIFESP), between 2007 and 2020. This database includes 1,000 pregnancies of women with GDM, with 17 women having been pregnant twice during the study. Inclusion criteria were women with ≥18 years old and fasting glucose >92 mg/dL and <126 mg/dL and/or abnormal oral glucose tolerance test with 75 g of dextrose, with fasting glucose >92 mg/dL and/or 1 h >180 mg/dL and/or 2 h >153 mg/dL, according to the diagnostic criteria suggested by The International Association of the Diabetes and Pregnancy Study Groups and the Brazilian Diabetes Society.^8^ Exclusion criteria for the current analysis were children under 2 years of age, children on continuous oral glucocorticoid medication, or those who used glucocorticoids during the period of data collection.

Women and their children were invited to participate in the study via phone contact, following standardized text templates, starting in November 2020. Of these, 223 women and 229 children responded and attended the appointments (92 refused, 49 missed the appointment, 306 had invalid contact, 311 did not respond to the contact, 6 were excluded due to fetal death, and 7 were excluded due to neonatal death). Additionally, two participants were excluded from the analyses because they did not have TG or HDL-cholesterol values, totaling 227 children. The children and the mother or legal guardians were scheduled for an in-person meeting at the Diabetes Center- Federal University of São Paulo to sign the informed consent form (ICF) to authorize the use of previously collected data during routine prenatal follow-up, as well as consent to participate in the collection of new in-person data, including interviews, physical exams, and laboratory tests of the women and their children.

During the in-person evaluation, all participants underwent a standardized questionnaire administered by the responsible researcher, which included data on identification, ethnicity, education, family income, personal and family health history, breastfeeding duration, lifestyle habits, physical activity, dietary quality, sexual maturation questionnaire, and vaccination records.

A physical examination was performed, including anthropometric measurements (weight, height, abdominal and cervical circumferences, and blood pressure measurements) with calibrated equipment and anatomical standardization. All participants were inspected in the cervical and axillary regions to check for signs of insulin resistance (acanthosis and acrochordons). Peripheral venous access and blood sample collection were performed at the units of the Diabetes Center of the Paulista School of Medicine and the Research Incentive Fund Association (AFIP) by trained professionals on previously scheduled dates between the researcher, participants, and units. All participants and legal guardians received instructions on the laboratory sample collection and appropriate fasting duration. The test used to assess glucose intolerance in the offspring included serum measurements of glycated hemoglobin, fasting glucose, and insulin levels. Participants received feedback on their test results, and when abnormalities were found, they were instructed on how to seek professional help.

Biochemical analyses were performed at the AFIP laboratory. The insulin reserve of the offspring was estimated through serum insulin and c-peptide levels measured by the electrochemiluminescence assay. Plasma glucose levels were assessed by a single fasting measurement determined using the glucose oxidase method; glycated hemoglobin was measured using an assay method certified by the National Glycohemoglobin Standardization Program. Total cholesterol and lipoprotein fractions were analyzed using the enzymatic colorimetric method, while triglycerides (TG) were measured by the enzymatic colorimetric peroxidase method. Insulin resistance was estimated using the HOMA-IR index (homeostasis model assessment-insulin resistance: fasting insulin mUI/mL/fasting glucose mmol/L×22.5).^9^ Blood was collected through venous puncture by trained professionals, and in case of failure, up to two attempts per patient were allowed, or the procedure was stopped if the patient or guardian did not consent to another attempt. The team ensured that the minimum necessary amount of samples for aliquoting was collected. The tubes contained the anticoagulant specified by the manufacturer. Each tube with the collected sample was labeled with patient data and the collection time. Transport packaging was done in triple packaging (primary leak-proof container, secondary leak-proof packaging, and external rigid packaging with adequate resistance for its capacity). To control the temperature, recyclable ice was used.

The exposure variable was the TG/HDL ratio. It was divided into tertiles, with the following cutoff points according to age groups: 2–9 years: 1.2 and 2.0; 10–14 years: 1.4 and 3.0.

Outcome variables were the cardiometabolic risk factors (body mass index [BMI], abdominal circumference, blood pressure, and blood glucose), insulinemia, and HOMA-IR. HOMA-IR was calculated using the formula: fasting glucose in mmol/L × fasting insulin in mUI/L/22.5.^9^ The cardiometabolic risk factors were defined according to WHO and Brazilian Society of Pediatrics criteria:^10,11^ BMI: For ≥5 years: Z-score ≥+2: obesity; ≥+1 and <+2: overweight; ≥-2 and <+1: normal; ≥-2: underweight; for <5 years: Z-score ≥+3: obesity; ≥+2 and <+3: overweight; ≥-2 and <+2: normal; ≥-2: underweight.

Abdominal circumference: elevated above the 90th percentile for those over 6 years old.^12^

Blood pressure (BP): BP ≥90 and <95: elevated; BP ≥95: hypertension.^10^

Glucose tolerance: Fasting glucose ≥100 mg/dL, HbA1c ≥5.7%=dysglycemia.

Lipid profile: Low HDL-cholesterol ≤45 mg/dL and elevated TG ≥130 mg/dL for >10 years and ≥100 mg/dL for 2–9 years.^10^

### Statistical analysis

Continuous variables are presented as mean and standard deviation (normal distribution) or as median and interquartile range (non-normal distribution), and categorical variables as frequency and percentage. The sample was stratified according to tertiles of TG/HDL ratio. The variables of interest were compared according to the TG/HDL ratio tertiles using analysis of variance (ANOVA) for continuous variables (for non-normally distributed variables, a logarithmic transformation was applied) or chi-square test for categorical variables. Comparison of the frequencies of the cardiometabolic risk factors according to the tertile of TG/HDL ratio was performed by chi-square test, assessing the p-value for the comparison of the three groups and the p-value of linear-by-linear to identify a linear trend across the tertiles. Comparisons were made for each age group stratum: 2–4 years; 5–9 years; 11–14 years. Linear regression (dependent variable HOMA-IR) was used to assess the association between TG/HDL ratio and the insulin resistance marker, adjusted for sex and BMI. The analysis was conducted using the Statistical Package for the Social Sciences^®^ program, version 16.0 (SPSS Inc., 2000). The significance level was set at 5%.

## RESULTS

**Figure 1:**
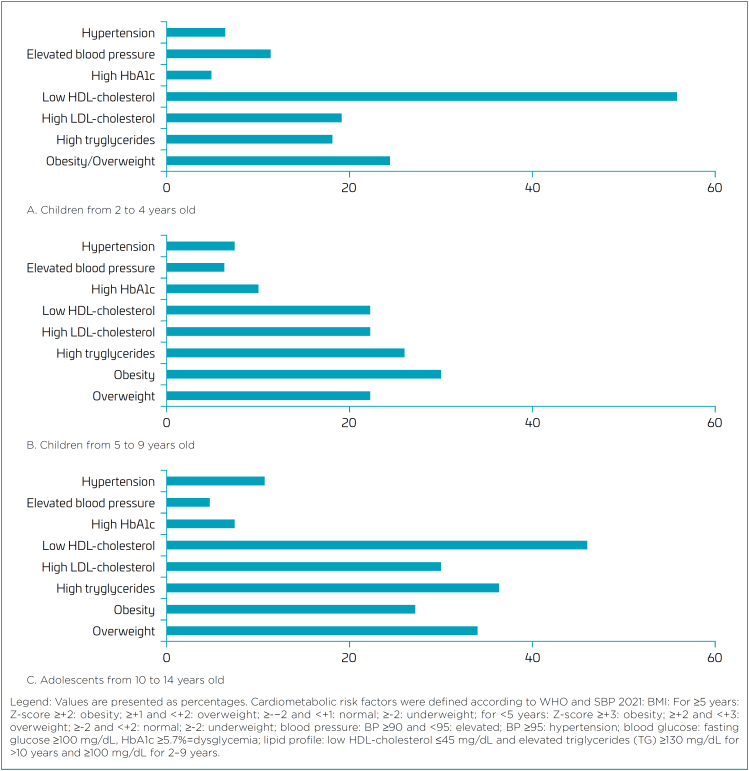
Prevalence of cardiometabolic risk factors in offspring from mothers who had gestational diabetes according to age range: 2–4 years; 5–9 years; 10–14 years.

A total of 227 children and adolescents were divided into three different age groups: 82 children aged 2–4 years, 118 children aged 5–9 years, and 27 adolescents aged 10–14 years. Figure 1 shows the prevalence of cardiometabolic risk factors in offspring from mothers who had GDM, according to age range: 2–4 years; 5–9 years; 10–14 years.

These age groups exhibited different medians and interquartile ranges for the TG/HDL ratio. The TG/HDL ratio was initially divided into tertiles with specific cutoff points for each age group. However, the values obtained for the 2–4 years and 5–9 years groups were very similar, prompting the decision to combine these children, distinguishing them from older children, who naturally have higher TG/HDL ratios due to physiological factors related to puberty. The following cutoff points were obtained: 2–9 years: 1.2 and 2.0; 10–14 years: 1.4 and 3.0.

In Table 1, we observed that in the 2–4 years age group, there was a statistical difference between the means according to the tertiles for the following characteristics: age, weight, height, HDL-cholesterol, low-density lipoprotein (LDL)-cholesterol, very-low-density lipoprotein (VLDL)-cholesterol, and TG; with borderline statistical significance for HbA1c. In the 5–9-year age group, there was a statistical difference between the means according to the tertiles for the following characteristics: weight, BMI, abdominal circumference, HbA1c, HDL-cholesterol, VLDL-cholesterol, TG, HOMA-IR, fasting insulin, and C-peptide; and statistically borderline for LDL-cholesterol, cervical circumference. In the 10–14 years age group, there was a statistically significant difference in means according to the tertiles was observed for the following characteristics: abdominal circumference, HDL-cholesterol, VLDL-cholesterol, and TG; and statistically borderline for weight, height, cervical circumference, HOMA-IR, fasting insulin, and C-peptide.

**Table 1:** Clinical characteristics of the children of mothers who had gestational diabetes according to the tertiles of triglycerides/high-density lipoprotein-cholesterol ratio, presented by age group.

Tertiles of TG/HDL ratio		2–4 years (n=82)	5–9 years (n=118)	10–14 years (n=27)
Age (years)				
	T1	3.0 (0.8)	7.0 (1.3)	10.6 (0.5)
	T2	2.4 (0.6)^a^	7.2 (1.5)	11.3 (0.9)
	T3	2.9 (0.8)^b^	7.4 (1.4)	11.2 (1.4)
	p-value	0.006	0.557	0.221
Birth weight (kg)				
	T1	3.3 (0.4)	3.2 (0.4)	3.2 (0.4)
	T2	3.0 (0.7)	3.2 (0.4)	3.2 (0.5)
	T3	3.2 (0.6)	3.3 (0.4)	3.0 (0.6)
	p-value	0.179	0.481	0.648
Weight (kg)				
	T1	16.3 (2.6)	28.4 (9.1)	40.5 (13.8)
	T2	14.0 (2.0)^a^	31.7 (11.9)	32.9 (13.3)
	T3	15.5 (3.9)	38.1 (13.9)^a^	55.0 (11.4)
	p-value	0.022	0.001	0.053
Height (m)				
	T1	1.0 (0.1)	1.3 (0.1)	1.5 (0.1)
	T2	0.9 (0.1)^a^	1.3 (0.1)	1.5 (0.1)
	T3	1.0 (0.1)	1.3 (0.1)	1.5 (0.1)
	p-value	0.001	0.111	0.086
BMI (kg/m^2^)				
	T1	16.1 (1.3)	17.4 (3.5)	18.8 (4.7)
	T2	16.3 (1.3)	18.8 (4.3)	22.3 (5.2)
	T3	16.4 (2.0)	21.4 (5.2)^a,b^	23.5 (5.1)
	p-value	0.726	<0.001	0.136
Systolic blood pressure (mmHg)				
	T1	88.5 (8.7)	92.8 (12.8)	98.4 (7.5)
	T2	85.4 (10.5)	93.8 (14.9)	99.3 (15.1)
	T3	87.2 (19.7)	93.1 (13.1)	99.9 (7.3)
	p-value	0.733	0.951	0.958
Diastolic blood pressure (mmHg)				
	T1	56.4 (9.0)	57.5 (9.4)	65.7 (5.7)
	T2	52.9 (7.3)	56.6 (10.5)	67.7 (11.7)
	T3	54.2 (13.0)	58.8 (8.7)	67.2 (9.1)
	p-value	0.476	0.619	0.889
Abdominal circumference (cm)				
	T1	51.6 (3.4)	63.0 (11.7)	68.1 (13.1)
	T2	49.8 (3.2)	66.1 (13.6)	79.1 (12.9)
	T3	52 (6.3)	71.8 (12.6)^a^	83.7 (13.2)^a^
	p-value	0.186	0.008	0.050
Neck circumference (cm)				
	T1	24.9 (1.8)	28.3 (2.5)	29.7 (2.8)
	T2	25.0 (2.7)	28.9 (3.2)	32.3 (4.0)
	T3	25.2 (1.8)	29.8 (3.0)	32.3 (3.3)
	p-value	0.896	0.069	0.087
Fasting glucose (mg/dL)				
	T1	82.6 (5.8)	82.1 (7.5)	81.8 (3.4)
	T2	82.3 (7.1)	83.6 (6.8)	85.6 (5.9)
	T3	83.0 (7.9)	83.6 (7.2)	84.8 (6.0)
	p-value	0.948	0.542	0.288
HbA1c (%)				
	T1	5.1 (0.2)	5.2 (0.3)	5.1 (0.3)
	T2	5.1 (0.3)	5.0 (0.5)	5.1 (0.3)
	T3	5.3 (0.4)^b^	5.3 (0.5)^b^	5.3 (0.4)
	p-value	0.052	0.033	0.275
Total cholesterol (mg/dL)				
	T1	149.9 (31.6)	164.7 (21.8)	173.6 (23.4)
	T2	144.0 (24.3)	173.7 (34.3)	168.6 (23.5)
	T3	159.8 (30.0)	162.7 (28.8)	157.2 (16.1)
	p-value	0.114	0.209	0.268
HDL-cholesterol (mg/dL)				
	T1	55.3 (12.4)	60.8 (10.1)	58.6 (11.1)
	T2	43.9 (8.6)^a^	53.5 (8.8)^a^	50.7 (7.8)^a^
	T3	38.4 (6.2)^a^	42.4 (8.2)^a,b^	34.8 (6.1)^a,b^
	p-value	<0.001	<0.001	<0.001
LDL-cholesterol (mg/dL)				
	T1	89.6 (19.3)	93.1 (18.0)	103.8 (16.4)
	T2	85.4 (21.7)^a^	103.9 (29.0)	98.2 (18.4)
	T3	101.2 (28.4)	94.9 (19.7)	82.4 (28.1)
	p-value	0.037	0.082	0.115
VLDL-cholesterol (mg/dL)				
	T1	10.6 (2.0)	12.8 (13.5)	11.2 (2.2)
	T2	14.4 (4.4)^a^	16.8 (4.0)	19.7 (6.1)^a^
	T3	20.2 (4.7)^a,b^	23.2 (6.6)^a,b^	40.0 (19.8)^a,b^
	p-value	<0.001	<0.001	<0.001
Triglycerides (mg/dL)				
	T1	52.7 (9.8)	54.1 (11.1)	56.1 (12.1)
	T2	69.3 (12.1)^a^	82.2 (18.0)^a^	98.8 (30.0)^a^
	T3	1007 (23.3)^a,b^	118.4 (30.5)^a,b^	199.8 (99.1)^a,b^
	p-value	<0.001	<0.001	<0.001
HOMA-IR*				
	T1	0.87 (0.68–1.15)	1.14 (0.75–1.78)	1.82 (1.62–2.36)
	T2	0.73 (0.48–0.97)	1.90 (0.90–2.64)	2,28 (1.57–4.11)
	T3	0.78 (0.56–1.57)	1.98 (1.27–2.75)^a^	4.35 (1.37–6.32)
	p-value	0.479	0.002	0.084
Fasting insulin (mU/l)*				
	T1	4.5 (3.3–1.4)	5.4 (2.7–8.7)	9.0 (8.2–11.6)
	T2	3.5 (2.3–5.0)	9.2 (4.4–12.4)	10.4 (7.6–19.3)
	T3	4.0 (2.8–8.0)	9.8 (6.0–13.6)^a^	21.0 (6.7–29.3)
	p-value	0.472	0.002	0.092
C-peptide C (ng/mL)*				
	T1	1.2 (0.8–1.4)	1.2 (0.9–1.7)	1.88 (1.4–2.3)
	T2	1.0 (0.8–1.3)	1.8 (1.1–2.6)^a^	2.6 (1.7–4.1)
	T3	1.2 (0.9–1.5)	1.9 (1.4–2.5)^a^	3.2 (1.7–4.2)
	p-value	0.434	<0.001	0.057

Values are presented as mean (standard deviation) and/or for variables with non-normal distribution

(*) as median (interquartile range). ANOVA was used for comparison. For non-normally distributed variables, logarithmic transformation was applied for use in ANOVA. Bonferroni corrections are presented when p<0.05: a vs. T1, b vs. T2.

HOMA-IR: fasting glucose in mmol/L × fasting insulin in mUI/L/22.5; BMI: body mass index.

In Table 2, we observed that there were no differences regarding characteristics of the variables collected during the antenatal period according to the current tertile of TG/HDL of the children.

**Table 2:** Maternal characteristics during pregnancy according to offsprings’ actual tertiles of triglycerides/high-density lipoprotein-cholesterol.

Variable		Tertile 1 TG/HDL	Tertile 2 TG/HDL	Tertile 3 TG/HDL	p-value
Age (years)		34.0 (5.7)	34.0 (6.1)	34.3 (5.0)	0.913
Pre-gestational BMI (kg/m^2^)		29.1 (5.9)	30.3 (6.5)	29.9 (5.7)	0.440
Pre-gestational obesity, n(%)		28 (36.4)	31 (43.7)	36 (48.0)	0.341
History of hypertension, n(%)		18 (23.4)	17 (23.6)	10 (13.5)	0.217
Fasting glucose in 1st trimester (mg/dL)		100.3 (9.0)	108.2 (6.9)	98.4 (5.6)	0.107
OGTT (2nd or 3rd trimester)					
	Fasting glucose (mg/dL)	98.1 (18.8)	97.3 (12.5)	101.2 (22.8)	0.571
	1 h glucose (mg/dL)	184.7 (37.1)	182.7 (34.6)	174.3 (45.8)	0.490
	2 h glucose (mg/dL)	152.9 (32.7)	159.8 (40.4)	158.9 (35.6)	0.594
Use of insulin during pregnancy, n (%)		30 (38.0)	25 (34.7)	34 (44.7)	0.500
Hypertension in pregnancy, n (%)		3 (4.1)	6 (9.0)	3 (4.5)	0.395
Birth weight according to gestational age					
	Small for gestational age	4 (5.4)	7 (10.1)	5 (6.8)	0.552
	Adequate for gestational age	67 (90.5)	57 (82.6)	62 (83.8)	
	Large for gestational age	3 (4.1)	5 (7.2)	7 (9.5)	

Values are mean (SD) or n (%). ANOVA or χ^2^ test was used as appropriate.

TG/HDL: triglycerides/high-density lipoprotein-cholesterol; BMI: body mass index.

In Table 3, according to the tertiles of TG/HDL ratio, we observed some differences in the prevalence of cardiometabolic risk factors. In the children with 2–4 years, there was a difference in the frequencies of elevated HbA1c, TG, and reduced frequencies of low HDL-cholesterol according to the tertiles. In those from 5–9 years, there was a difference in the frequencies of low HDL-cholesterol, elevated TG, and overweight or obesity, with a progressive increase in the frequency of TG, overweight, and obesity and a reduction in the frequencies of low HDL-cholesterol according to the tertiles.

**Table 3:** Frequencies of cardiometabolic risk factors in children of mothers who had gestational diabetes according to the tertiles of triglycerides/high-density lipoprotein-cholesterol ratio, presented by age group.

Tertiles of TG/HDL ratio		2–4 years (n=82)	5–9 years (n=118)	10–14 years (n=27)
Elevated HbA1c				
	T1	0	5 (10.9)	1 (11.1)
	T2	1 (3.7)	5 (13.9)	0 (0.0)
	T3	4 (12.9)	3 (8.6)	1 (11.1)
	p χ^2^ test	—	0.465	0.538
	p linear-by-linear	<0.001	0.920	1.000
Low HDL-cholesterol				
	T1	4 (16.7)	1 (2.2)	2 (22.2)
	T2	14 (51.9)	3 (8.3)	2 (22.2)
	T3	27 (87.1)	23 (65.7)	8 (88.9)
	p χ^2^ test	<0.001	<0.001	0.005
	p linear-by-linear	<0.001	<0.001	0.005
Elevated triglycerides				
	T1	0	0	1 (11.1)
	T2	1 (3.7)	8 (22.2)	2 (22.2)
	T3	14 (45.2)	25 (71.4)	7 (77.8)
	p χ^2^ test	—	—	0.007
	p linear-by-linear	<0.001	<0.001	0.004
High LDL-cholesterol				
	T1	4 (16.7)	8 (17.4)	5 (55.6)
	T2	2 (7.4)	12 (33.3)	2 (22.2)
	T3	10 (33.3)	7 (20.0)	1 (11.1)
	p χ^2^ test	0.044	0.206	0.099
	p linear-by-linear	0.101	0.684	0.043
Overweight or obesity				
	T1	6 (25.0)	18 (39.1)	3 (33.3)
	T2	8 (29.6)	17 (47.2)	6 (66.6)
	T3	9 (30.0)	27 (75.0)	7 (77.7)
	p χ^2^ test	0.907	0.007	0.284
	p linear-by-linear	0.696	0.001	0.044
Elevated blood pressure or hypertension				
	T1	4 (16.7)	7 (15.2)	0 (0.0)
	T2	4 (14.8)	5 (14.3)	2 (22.2)
	T3	8 (27.6)	3 (8.4)	2 (22.2)
	p χ^2^ test	0.331	0.816	0.361
	p linear-by-linear	0.707	0.537	0.281

Values are presented as frequency (percentage). The chi-square test was used to compare the frequencies between the TG/HDL tertile groups. Cardiometabolic risk factors were defined according to WHO and SBP 2021: BMI: for ≥5 years: Z-score ≥+2: obesity; ≥+1 and <+2: overweight; ≥-2 and <+1: normal; ≥-2: underweight; for <5 years: Z-score ≥+3: obesity; ≥+2 and <+3: overweight; ≥-2 and <+2: normal; ≥-2: underweight; blood pressure: BP ≥90 and <95: elevated; BP ≥95: hypertension; blood glucose: fasting glucose ≥100 mg/dL, HbA1c ≥5.7%=dysglycemia; lipid profile: low HDL-cholesterol ≤45 mg/dL and elevated triglycerides (TG) ≥130 mg/dL for >10 years and ≥100 mg/dL for 2–9 years.

TG/HDL: triglycerides/high-density lipoprotein-cholesterol; BMI: body mass index.

For those from 10 to 14 years, there was a difference in the frequencies of low HDL-cholesterol and elevated TG, with a progressive increase in the frequency of LDL-cholesterol, TG, overweight, and obesity, and a reduction in the frequency of low HDL-cholesterol according to the tertiles.

**Figure 2:**
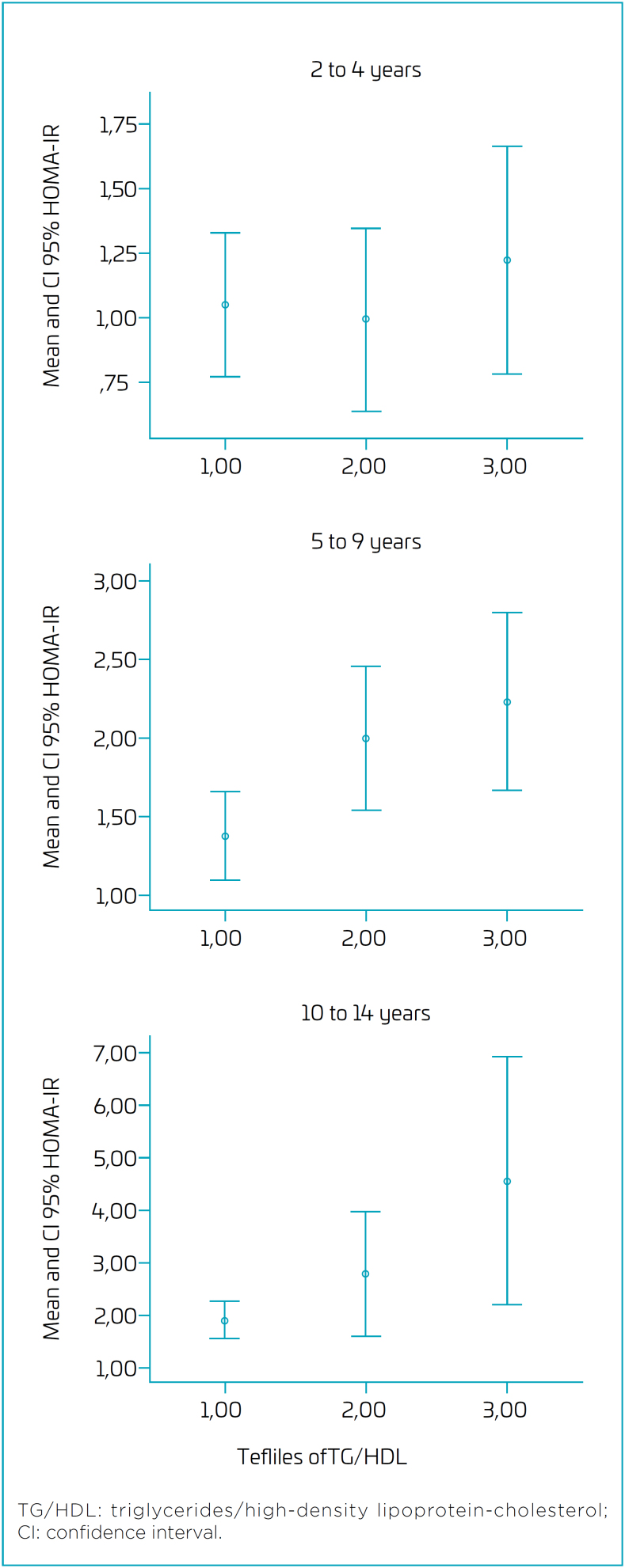
Mean and 95%CI of HOMA-IR in children of mothers with gestational diabetes according to triglycerides/high-density lipoprotein-cholesterol ratio tertiles for each age group.

In Figure 2, we observe an increase in the mean (95%CI) of HOMA-IR according to the TG/HDL ratio tertiles, particularly in the 5–9 years and 10–14 years age groups.

In the linear regression, considering HOMA-IR (log) as the dependent variable, we observed a statistically significant association with the TG/HDL ratio variable for the age groups of 5–9 years and 10–14 years, even after adjusting for sex and BMI of the children (Table 4). The adjustment with waist circumference instead of BMI did not alter the results (data not shown). For children aged 2–4 years, we did not observe a significant association between TG/HDL ratio and HOMA-IR, either in the unadjusted model or after adjusting for sex and BMI or waist circumference.

**Table 4:** Association between homeostasis model assessment of insulin resistance (log) and triglycerides/high-density lipoprotein-cholesterol ratio in children of mothers with gestational diabetes according to age group.

Years	Crude model Beta (95%CI)	p-value	Adjusted model Beta (95%CI)	p-value
2–4	0.04 (-0.14–0.21)	0.693	0.02 (-0.16–0.20)	0.840
5–9	0.22 (0.10–0.34)	<0.001	0.11 (0.00–0.23)	0.046
10–14	0.13 (0.06–0.20)	0.001	0.10 (0.02–0.18)	0.017

Model adjusted for sex.

BMI: body mass index; CI: confidence interval; HOMA-IR: homeostasis model assessment of insulin resistance; TG/HDL: triglycerides/high-density lipoprotein-cholesterol.

## DISCUSSION

The current study shows a worse cardiometabolic profile and higher HOMA-IR levels according to TG/HDL ratio terciles in children aged 2–14 years born to mothers with GDM, regardless of sex and BMI. This supports our hypothesis that the TG/HDL ratio could be a useful marker of insulin resistance syndrome in this population.

This is especially relevant given rising obesity rates and worsening cardiometabolic profiles in children worldwide,^13,14^ reinforcing the need for early interventions. Pregnancies complicated by GDM increase the offspring's risk of obesity and related conditions,^15,16^ making early identification crucial. This study contributes by evaluating the TG/HDL ratio as a marker of cardiometabolic risk in children of mothers with GDM.

Regarding the prevalence of cardiometabolic risk factors in the children of mothers with GDM, the values found are higher than those from national and international studies for the same age groups. We observed a higher prevalence of obesity in our sample when compared to the POF (Pesquisa de Orçamento Familiar) 2008–2009,^17^ which showed 16.6% obesity in boys and 11.8% in girls in the 5–9 years age group, and 5.9% in boys and 4.0% in girls in the 10–14 years age group, while in our sample, these rates of overweight or obesity were 28% in children aged 2–4 years, 30% in children aged 5–9 years, and 25.9% in children aged 10–14 years (Figure 1). Our results also show higher obesity rates than those presented by the World Obesity Federation's 2023 Atlas for the Americas region (North, Central, and South America), which were 20% for boys and 16% for girls aged 5–19 years in 2020.^15^ The ERICA,^1^ a nationwide school-based multicenter survey involving over 70,000 adolescents aged 12–17 from Brazilian cities with more than 100,000 inhabitants, provides important data on the prevalence of cardiometabolic risk factors in the 10–19 years age group. The main findings of the study showed prevalences of overweight/obesity at 25.5%, elevated blood pressure at 9.6%, low HDL-cholesterol at 47.3%, hypertriglyceridemia at 7.8%, and elevated blood glucose at 4.0%.^1^ In comparison to this study, our sample showed higher prevalences of all cardiometabolic risk factors in the 10–14 years age group, with prevalences of 33.3% for overweight, 25.9% for obesity, 3.7% for elevated blood pressure, 11.1% for systemic hypertension, 44.4% for low HDL-cholesterol, 37% for hypertriglyceridemia, and 7.4% with elevated HbA1c. These results confirm the cardiometabolic risk profile of this population of children born to mothers with GDM, reinforcing the impor-tance of investigating risk markers, as done in the current study.

In the current study, we showed the association of TG/HDL ratio with both serum glucose levels, lipid profile, BMI, waist and neck circumference, and HOMA-IR, as well as with the frequencies of some of the cardiometabolic risk factors, such as elevated HbA1c and overweight or obesity, in children and adolescents of mothers with GDM, especially in children aged 5–14 years. The use of TG/HDL ratio as a cardiometabolic risk marker has gained attention due to intriguing results in different populations. In adults, the TG/HDL ratio has gained attention due to evidence of its association with metabolic syndrome, insulin resistance, cardiovascular events, and diabetes.^18-21^ Studies in children and adolescents on this topic are scarcer and mainly focus on specific groups such as youth with obesity. These studies have positive conclusions about the direct association of TG/HDL ratio with obesity and insulin resistance, but the population sample has typically consisted of children who are already considered obese and over 10 years old.^5,6,9,22^ In this context, our findings suggest that the TG/HDL ratio may be a useful marker for children and adolescents, offspring of mothers with GDM, even without obesity, and in younger age groups, such as those aged 5–9 years.

It is important to note that in children, cardiometabolic changes such as hypertension or diabetes may not yet be evident, which may explain the lack of significant differences between TG/HDL ratio terciles. This highlights the value of early markers that can identify children undergoing unfavorable pathophysiological changes potentially leading to diabetes, hypertension, dyslipidemia, and long-term cardiovascular risk.

The association between cardiometabolic profile and insu-lin resistance is well established,^23,24^ which supported our eval-uation of TG/HDL ratio in relation to HOMA-IR levels. TG and HDL-cholesterol are easily, routinely, and cost-effec-tively measured compared to insulin, making them useful for predicting insulin resistance. In this context, Dr. Reaven, who introduced the concept of insulin resistance syndrome (later metabolic syndrome), concluded that a high TG/HDL ratio may be as effective as metabolic syndrome classification in identifying insulin resistance and predicting cardiovascular diseases.^22^ Our findings support this, as HOMA-IR levels increased with TG/HDL ratio terciles, and linear regression showed a significant association for the 5–9 and 10–14 years age groups, even after adjustments for sex and BMI. This reinforces the potential of TG/HDL ratio as a pediatric cardiometabolic risk marker. Besides its positive associations, TG/HDL ratio is a practical marker due to its low cost and routine use.^18-20^

Our study has limitations, such as sample size in the 10–14 years group, which may have influenced the lack of significant differences in some variables. Furthermore, the sample is based in children of mothers with gestational diabetes referred to a specialized service with multidisciplinary assistance, and well control of the disease. However, it is important to notice, in terms of generalizability, that at the time of the antenatal care collections, all women diagnosed with gestational diabetes in Brazil were referred to specialized care services. More recently, however, the Ministry of Health has revised this guideline, and currently only those requiring insulin therapy are referred for specialized follow-up. Although cross-sectional, the study offers the possibility of future follow-up to assess the predictive value of TG/HDL ratio over time. Its strength lies in the long-term follow-up of children of mothers with GDM, which is a rare approach in the literature, particularly in Brazil. These women were monitored during pregnancy, and data were collected in prenatal care, ensuring diagnostic accuracy and avoiding memory bias. Currently, 2–14 years post-delivery, mothers and children were evaluated using a standardized and rigorous protocol. We also performed an additional analysis evaluating selected maternal characteristics during pregnancy according to the offspring TG/HDL tertiles (assessed 2–14 years after delivery), and no significant differences were observed. This suggests that the severity of GDM during pregnancy did not differ across tertiles.

In conclusion, the current study shows that the TG/HDL ratio can identify children of mothers with GDM, aged 2–14 years, with worse cardiometabolic profiles and higher insulin resistance indices. The TG/HDL ratio can be widely used in clinical practice and may help guide the search for insulin resistance syndrome and the appropriate follow-up of children and adolescents at higher risk for developing diabetes and cardiovascular disease over the years.

## Data Availability

The database that originated the article is available with the corresponding author. CAAE: 68148823.5.0000.5505
